# The Interplay between Antiviral Signalling and Carcinogenesis in Human Papillomavirus Infections

**DOI:** 10.3390/cancers12030646

**Published:** 2020-03-10

**Authors:** Ana Rita Ferreira, Ana Catarina Ramalho, Mariana Marques, Daniela Ribeiro

**Affiliations:** Institute of Biomedicine—iBiMED & Department of Medical Sciences, University of Aveiro, 3810-198 Aveiro, Portugal; arferreira@ua.pt (A.R.F.); ana.catarina.ramalho@ua.pt (A.C.R.); mar.marques@ua.pt (M.M.)

**Keywords:** human papillomavirus, innate immunity, cancer, intracellular antiviral response, immune evasion

## Abstract

Human papillomaviruses (HPV) are the causative agents of the most common sexually transmitted infection worldwide. While infection is generally asymptomatic and can be cleared by the host immune system, when persistence occurs, HPV can become a risk factor for malignant transformation. Progression to cancer is actually an unintended consequence of the complex HPV life cycle. Different antiviral defence mechanisms recognize HPV early in infection, leading to the activation of the innate immune response. However, the virus has evolved several specific strategies to efficiently evade the antiviral immune signalling. Here, we review and discuss the interplay between HPV and the host cell innate immunity. We further highlight the evasion strategies developed by different HPV to escape this cellular response and focus on the correlation with HPV-induced persistence and tumorigenesis.

## 1. Introduction

Human papillomaviruses (HPV) are the main causative agents of cervical cancer and represent the most common sexually transmitted infection worldwide [1,2]. To this date, over 200 HPV types have already been identified [2]. HPV infections are usually asymptomatic and cleared by the immune system within 12 months [3]. However, HPV-infected immunocompromised individuals are susceptible to the development of HPV-associated carcinomas and, depending on the HPV type, infection can be a major risk factor for malignant progression [1]. High-risk HPV (HR-HPV) types are associated with the development of several other carcinomas, such as anal, vulvovaginal and penile, head and neck cancers [1,3,4].

The innate immune system is an early defence mechanism triggered upon detection of pathogens, such as viruses [5]. The efficient activation of the immune response is the key between viral clearance and viral persistence. Upon infection, the recognition of essential viral components, named pathogen-associated molecular patterns (PAMPs), by the cellular pattern-recognition receptors (PRRs) leads to the activation of the innate immune response and, ultimately, the adaptive immune response [6].

In general, cellular PRRs can detect either viral RNA or DNA, and they can be either associated with membranes or localize freely in the cytosol [7,8]. These different classes of PRRs use common pathways to convey their signals, ultimately culminating in the expression of pro-inflammatory cytokines, such as type I interferons (IFNs), and IFN-stimulated genes (ISGs), restricting infection establishment and spreading [9]. This is accomplished by triggering the activation of downstream signalling pathways, namely the IFN-regulatory factors (IRFs) pathways, the janus kinase/signal transducers and activators of transcription (JAK-STAT) pathway and the nuclear factor-κB (NF-κB) signalling pathway [6,10].

Understanding the biology of HPV infection was only possible in the last few decades with the advent of molecular cloning and the development of organotypic cultures, allowing not only the study of individual viral genes but also the analysis of viral infections and their progression. Nevertheless, there is still a gap in the knowledge concerning the interplay between innate immune evasion and cancer progression during HPV infection. Here, we review and clarify these different evasion mechanisms and discuss their correlation with cancer progression during infection.

## 2. Human Papillomavirus Biology

HPV can infect cutaneous epithelial cells or mucosal tissues and, depending on their tropism, HPV are categorized either as cutaneous or mucosal. Additionally, HPV can be further divided into two categories: Low-risk HPV (LR-HPV), which cause benign warts, and HR-HPV with oncogenic potential (Table 1) [1,2,4,11].

**Figure 1:**
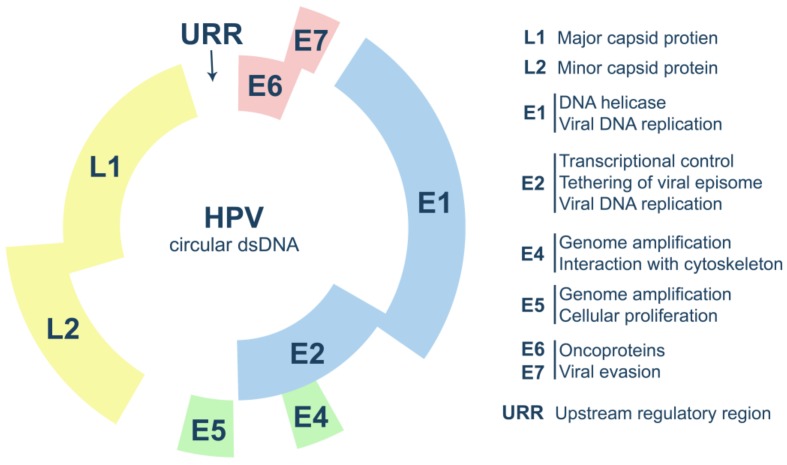
HPV genome organization and functions of the main viral proteins.

HPV particles are composed by a non-enveloped icosahedral capsid that shields the viral genome. The HPV genome consists of a single circular double-stranded DNA (dsDNA) molecule associated with host-derived histones, and, typically, encodes seven to eight open reading frames (ORFs) (Figure 1).

The genome’s main structural and organizational features are shared, with some variations, among different HPV types [12,13]. The genome is divided into three functional regions: Early (E), late (L) and the upstream regulatory region (URR) (Figure 1). The E region is composed of six ORFs that codify the proteins E1, E2, E6, E7, E4 and E5. Additionally, it has been reported that some HPV can also express E3, E8 and an ‘E5 like protein’ also known as E10 [13,14]. The L region encodes for the structural proteins L1 and L2. The URR (previously known as long control region or LCR) corresponds to a non-coding segment containing the cis elements essential for viral replication and transcription by the host machinery [2,13]. E1, E2, L1 and L2 have well-conserved sequences, while the remaining genes display a greater variability, resulting in the differences observed during infections with the different HPV types [15].

### 2.1. HPV Life Cycle

**Figure 2:**
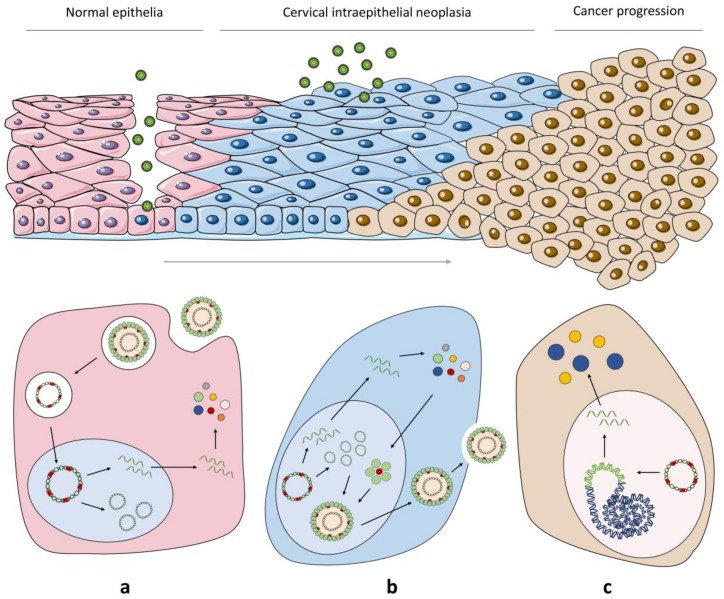
HPV life cycle and progression to cancer. (**a**) HPV reaches the basal cells of the epithelium through tissue abrasions. Upon recognition, endocytosis of the virion occurs, and HPV is transported through the endosomal pathway. At endosomes, L2 mediates viral egressing and HPV vesicles are transported along microtubules to the nucleus, where early transcription is initiated, with a quick but transient expression of the early proteins and through the recruitment of the cellular DNA replication machinery. Afterwards HPV can enter the latency phase. (**b**) Once basal cells start to differentiate, they migrate towards the surface of the tissue. Here, structural proteins are expressed, allowing virion assembly and release, which occurs alongside with tissue desquamation. (**c**) Viral persistence in basal cells can result in HPV genome integration, which promotes cancer progression.

HPV infects the basal stem cells of the stratified epithelia (which are the only epithelial cells capable of undergoing cell division) of the skin, oral cavity and anogenital track [1,2]. As the basal layer of the epithelium is protected by several layers of differentiated cells, HPV reaches this area via micro wounds in the tissue (Figure 2a) [16,17].

Upon recognition of L1 at the cell surface, the viral capsid undergoes structural modifications, required for endocytosis of the virion [18,19,20]. Then, while HPV travels along the endosomal pathway, L1 dissociates from the viral genome and L2 mediates viral egressing from the endosomes, guiding HPV vesicles along microtubules [21,22,23]. When the nuclear envelope is disrupted during mitosis, L2-viral DNA complexes are delivered into the nucleus [17,24,25]. Upon nuclear entry, viral early transcription is initiated with the expression of the early proteins E1 and E2, essential factors for viral DNA replication, as they recruit the cellular DNA replication machinery [26,27]. Additionally, E2 is also critical for transcription, since it has splicing-regulating activities that control the processing of viral pre-mRNA [28,29,30]. E1, E2, E6 and E7 are the most early proteins and are found in the basal layers of the epithelium, while E5 and E4 only start to be detected at the suprabasal layers [11].

Three phases of replication in the viral life cycle have been identified: Initial amplification, maintenance replication and vegetative amplification (reviewed in [11,31,32]). Initially, replication starts at the URR, causing a quick but transient increase of viral genome copies (Figure 2a). Afterwards, viral DNA is stably maintained in low copy numbers during basal cells division. This is accomplished through the establishment of stable episomes at specific regions of the nucleus. E2 is then responsible for tethering the viral genome to host chromatin, thus guarantying its successful partition in equal amounts upon cell division [32]. HPV infection can persist in the latent form for years to decades until a switch from genome maintenance to vegetative viral replication occurs, allowing virion production (Figure 2b) [2,11].

In non-infected epithelia, the daughter cells from the proliferative basal layer lose contact with the basement membrane and stop dividing, initiating the process of terminal differentiation and ceasing their proliferative capacity [11,17]. However, as HPV is highly dependent on the host-cell machinery for viral genome replication and translation, it has evolved to carry out its replication cycle in concert with epithelial differentiation, alongside viral gene expression. HPV-infected cells retain their differentiation capacity and are capable of moving into the upper epithelial layers [11]. The molecular mechanisms behind this cell reprogramming are not yet well understood, but viral-encoded oncogenes seem to have a central role in this process. Moreover, vegetative amplification, besides being associated to an increase in HPV genome copy numbers, is also followed by the expression of the structural proteins L1 and L2 [2,11]. In the infected cells from the upper epithelium layers, virion assembly starts at the nucleus, where the capsid and genome packaging occur. Since HPV is a non-lytic virus, virions are only released when the infected cells reach the granular surface layers of the epithelium (Figure 2b) [11].

### 2.2. From HPV Infection to Malignant Transformation

Progression to cancer is a rare event in HPV infection. Indeed, this is an unwanted event for the virus, since infected cells that suffer transformation do not produce any virions. The mechanisms behind transformation of infected cells are not yet clearly understood, but it is known to be highly dependent on HR-HPV E6 and E7 oncogenes [33]. These proteins present a broad-spectrum functionality and modify different pathways, mainly associated to cell growth, differentiation and host genome stability (reviewed in [34]). It is thought that the trigger for cell transformation starts with the integration of viral episomes into the host genome (Figure 2c). In the reported cases, the viral genome is partially integrated occurring the loss of E2 ORF, which codifies the transcription repressor of the oncoproteins E6 and E7 genes [33,35,36]. It was also suggested that integration may occur as an indirect effect of episomes tethering, close to chromosomal regions that accumulate DNA break repair factors, which are required for viral genome replication [37].

Without the E2 repressor function, E6 and E7 start to be highly expressed during progression from cervical intraepithelial neoplasia (CIN) in HR-HPV infected tissues, contributing to a malignant evolution to invasive cancer [38,39,40]. Additionally, the integrated HPV genome seems to recruit DNA repair/recombination systems, which induce alterations in the host cell genome and eventually affect several genes, including cellular oncogenes that aid the transformation initiated by HPV oncoproteins. It was even suggested that de novo infections by new HPVs in cells that already present integrated HR-HPV genome can occur, potentiating the genomic instability [31,41].

The most important functions of E6 and E7 for carcinogenesis are the impairment of p53 and pRb tumour suppressor’s pathways, respectively [42]. Independently, E6 also stimulates the telomerase, which prevents senescence by stabilizing telomere length at the chromosomes’ ends [34,42]. E6 also contains a binding motif for PDZ (PSD-95/DLG/ZO-1) proteins, which induces the degradation or subcellular localization alterations of numerous cellular proteins. Both of these functions are assigned exclusively to HR-HPV E6 proteins [34].

## 3. Activation of the Antiviral Immune Signalling

The epithelial cells that constitute the cutaneous and mucosal tissues are the first line of defence of the innate immune system, and constitutively express low levels of IFNs and cytokines. Additionally, in the case of mucosal epithelia, cells produce mucin that prevents viral attachment and penetration [43]. HPV is able to bypass this protection through abrasions in the tissue [17].

**Figure 3:**
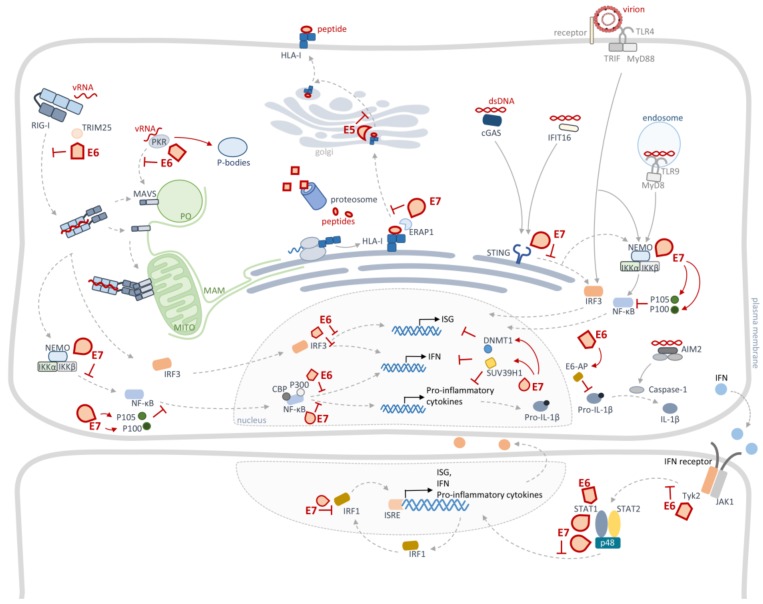
Evasion of the cellular innate immunity response by HPV proteins. HPV E2, E5, E6 and E7 target several steps of the pattern-recognition receptors (PRRs) signalling, downregulating the expression of interferons (IFNs), pro-inflammatory cytokines and IFN-stimulated genes (ISGs), and consequently inhibiting the cellular antiviral response, as well as antigen presentation at cell surface.

While viral infections are generally extremely immunogenic, the efficient HPV life cycle grants protection against epithelia defences. The hallmarks of HPV infection are the slow replication cycle and the virus capability to maintain low levels of viral proteins expression and secretion. Additionally, since HPV virions are only released at the epithelium surface without inducing cell lysis, no inflammatory response or viremia occur [1,12]. Thus, HPV is able to delay viral detection and elimination by the innate immune system. Nonetheless, to further ensure its ability to surpass immune surveillance, HPV has developed numerous strategies to evade and manipulate the cellular antiviral defences, by interfering with the function of host antiviral proteins or by inhibiting their expression (Figure 3).

Upon HPV infection, viral DNA is released into the cytosol and can be sensed by the PRRs IFN-inducible protein 16 (IFI16) [44,45], absent in melanoma 2 (AIM2) [46], and the toll-like receptors 4 (TLR4) [47] and TLR9 [48]. It has been reported that the activation of the cytosolic dsDNA sensors IFI16 and AIM2, induces the formation of the inflammasome complex that is required for processing and release of the pro-inflammatory cytokines interleukin-1β (IL-1β) and IL-18. Moreover, it was demonstrated that the ISG IFI16 ultimately restricts HPV replication by inducing epigenetic alterations on the viral genome [45].

Regarding the TLRs signalling, it has been shown that TLR4 is able to recognize the association of HPV11 with the heparin sulfate [49] or the glycosaminoglycans at the cell surface [49,50]. Additionally, it has been shown that TLR9 is able to recognize and be stimulated by the CpG motifs present in the HPV16 E6 gene sequence [48], during viral capsid disassembling in the endosome [20]. Induction of TLRs signalling pathways ultimately leads to the expression of IFNs and pro-inflammatory cytokines [51]. In fact, an association between the clearance of initial HPV infection in young women and higher expression levels of TLR3, TLR7, TLR8 and TLR9 has been reported [52,53]. Nevertheless, the signalling behind HPV genome recognition by TLRs remains to be elucidated.

It was reported that several cytokines, namely IL-1α, IL-4 IL-13, transforming growth factor β (TGF-β), tumor necrosis factor α (TNF-α), IFN-α and IFN-β, act as inhibitors of HPV16 URR activity, suppressing early gene transcription [54]. Furthermore, type I IFNs, TNF-α and TGF-β restrain the growth of non-infected and HPV-infected keratinocytes, while this suppression tends to cease in oncogenic cells [55,56]. Additionally, epithelial cells of cutaneous and mucosal tissues produce a single specific type I IFN, IFN-κ [57], which has been shown to have antiviral functions during HPV16 and HPV31 infection [58,59].

In addition to cytokines and ISGs, it was found that apolipoprotein B mRNA-editing catalytic polypeptide 3 (APOBEC3) proteins, an IFN-inducible antiviral family, promote hypermutations in the HPV genome [60], and reduces HPV infectivity [61]. Recently, the Myb-related transcription factor partner of profilin (MYPOP) was also shown to have antiviral activity against HPV by repressing the URR function [62]. Another important class of molecules that impede HPV infection are human α-defensins, immune system components that have broad antimicrobial effects [63]. Different mechanisms of inhibition have been proposed: For example, the α-defensin 5 (HD5) was reported to inhibit the furin cleavage of HPV L2, as well as to impede the dissociation of L1 from L2, which is essential for the entry process of HPV [64,65]. Moreover, it has been shown that human neutrophil peptides 1-4 (HNP1-4) and HD5 block escape from endocytic vesicles instead of inhibiting the binding or internalization in multiple serotypes of HPV infection [63,66]. Furthermore, defensins have been proposed to recruit immune cells, thus contributing to the activation of adaptive immunity [67].

These findings demonstrate that several immune effectors mount a response to HPV infection, eventually leading to viral clearance.

## 4. Cellular Innate Immunity Evasion by HPV

During its life cycle, HPV produces a small number of proteins, which therefore have multifunctional roles during infection. Curiously, HR-HPV oncoproteins E6 and E7 are the ones that interfere the most with the cellular innate immunity, alongside with minor roles of E5 and E2 (summarized in Figure 3 and Table 2).

Besides interfering with the cellular antiviral signalling pathways, HPV also impairs the antigen processing machinery, impeding T-cell recognition of infected cells. HPV directly impedes the generation of cytotoxic T-lymphocyte (CTL) epitopes through different mechanisms mediated by E7 and E5 [69,70,71,81]. HPV16 E7 also increases the expression of ERAP1, an immunopeptidase essential for epitope editing [81], which leads to a reduction of CD8+ T cell responses. It has furthermore been shown that the attenuation of ERAP1 induces CTL-mediated HPV-infected cell death [81]. Similarly, HPV16 E5 also impairs the transport of HLA-I to the cell surface [69,70,71]. In the last few years, new data on HPV epigenetic control has been emerging. Lo Cigno et al. showed that HPV E7 upregulates the H3K9 methyltransferase SUV39H1, which, through alterations in the chromatin structure, promotes epigenetic inhibition of nucleic acid sensors, such as RIG-I, cyclic GMP-AMP synthase (cGAS) or even STING [89].

### 4.1. HPV Targets Pattern Recognition Receptors

Several reports have shown different HPV strategies to directly impair PRRs signalling. It has been demonstrated that HPV16 and HPV18 E7 bind to STING, inhibiting the cGAS-STING signalling pathway [88]. STING is the adaptor protein that mediates the immune signalling upon recognition of viral DNA by a numerous set of cytosolic receptors, leading to the expression of type I IFNs and pro-inflammatory cytokines [95,96,97,98,99]. The role of STING in HPV infection recognition is still unclear and further studies should be performed.

Interestingly, it has been shown that HPV16 E6, but not E7, forms a complex with TRIM25 and its regulator ubiquitin carboxyl-terminal hydrolase 15 (USP15), inducing TRIM25 degradation [73]. TRIM25 is an E3 ubiquitin ligase essential for RIG-I activation, allowing the induction of its downstream signalling and consequential expression of IFNs and ISGs [100,101,102]. Whereas RIG-I only recognizes dsRNA, different studies have shown the activation and evasion of this signalling pathway by DNA viruses [103,104,105,106,107,108,109,110,111]. Thus, the role of RIG-I signalling in HPV sensing needs to be further investigated, but its targeting by E6 suggests that it plays a critical role on the HPV immune signalling.

### 4.2. HPV Targets Interferon Regulatory Factors Signalling

Several PRRs signalling pathways converge to activate the transcription factor IRF3, responsible for IFNs expression. As expected, HPV also targets this transcription factor to inhibit its translocation to the nucleus. It has been shown that HPV16 E6 binds to IRF3, although the same was not observed for HPV6 or HPV18 [74]. The IFN-κ induced IRF1 is another target of E7 from HPV16, HPV 18 and HPV11, resulting in the inactivation of its promoter activity [82,83,84,94]. It was proposed that IRF1 targeting prevents the correct binding to the IFN-β promoter region, in a mechanism that involves HDACs, leading to reduced IFN-β production [83]. Thus, IRFs targeting by HPV proteins leads to the impairment of IFN-α [79], IFN-β [74,83] and IFN-κ expression [112].

HPV16 was also reported to upregulate the ubiquitin carboxyl-terminal hydrolase L1 (UCHL1), to indirectly impair IRF signalling. This protein inhibits the K63 poly-ubiquitination of TNF receptor-associated factor 3 (TRAF3), suppressing IRFs activation [113].

### 4.3. HPV Targets NF-κBs Signalling

Another transcription promoter activated by PRRs signalling is the NF-κB. NF-κB signalling is a tightly regulated pathway that culminates on the regulation of several genes associated with immune and stress responses, as well as apoptosis, proliferation, differentiation and development (reviewed in [114,115]). HPV has evolved mechanisms to abrogate the immune and inflammatory response promoted by NF-κB signalling through E6 and E7.

When inactivated, NF-κB is complexed with its repressors and, upon activation, their degradation is induced in order to allow translocation of NF-κB to the nucleus [114,115]. It has been reported that HPV E7 associates with the inhibitor of IKK complex, impairing the release of NF-κB [75]. Furthermore, HPV16 E6 reduces the transcriptional activity of NF-κB, by interacting with its coactivators CREB binding protein (CBP) and p300 [75,76]. Interestingly, HPV6 E6 was also reported to bind the same coactivators, although less efficiently, and with a smaller inhibitory effect [76]. E7 was also reported to decrease the DNA binding activity of NF-κB and to reduce nuclear translocation and acetylation of the p65 subunit of NF-κB [82,116,117]. Furthermore, E7 was shown to bind to P/CAF, impairing its interaction with p65 [83], and to target the transcriptional coactivator p300 [85]. However, it has been demonstrated that the E2-dependent transcription requires CBP/p300. Thus, E7 interferes with the regulation of E2 transcriptional activity by associating with p300 [118]. It has also been reported that HPV16 E6 and E7 proteins induce the overexpression and modulate the subcellular localization of p105 and p100, NF-κB precursors [93].

As previously mentioned, HPV16 induces the overexpression of UCHL1 [113]. Binding of UCHL1 to TRAF6 leads to the degradation of NF-κB essential modulator (NEMO), which in turn results in the suppression of p65 phosphorylation, blocking the canonical NF-κB signalling [113]. UCHL1 also targets IκBα by attenuating its ubiquitination, preventing the release of NF-κB [119].

NF-κB signalling is a crucial mediator of inflammatory responses and regulates the expression of different interleukins [120]. Cellular inflammatory responses are also targets of HPV E6 and E7 proteins. It has been shown that E6 inhibits IL-18, a pro-inflammatory cytokine, by binding to its receptor [121,122]. E7 was also reported to bind and impair IL-18 receptor signalling [122]. Moreover, HPV16 E6 binds to the ubiquitin ligase E6-AP, inducing the degradation of pro-IL-1β in a proteasome-dependent manner, impairing IL-1β processing and secretion [72]. HPV also represses NF-κB-mediated transcription of AIM2, through overexpression of sirtuin 1 (SIRT1) [123].

While these studies propose an anti-inflammatory role for HPV E6 and E7, there is still some contradictions associated with the HPV effect over NF-κB signalling, since in vivo data suggests that HPV promotes chronic inflammation, which correlates with HPV-induced carcinogenesis [124,125].

### 4.4. HPV Targets JAK/STAT Signalling

E6, from HPV16 and HPV18 but not HPV11, directly interacts with TYK2 impairing binding with the transmembrane IFN-α/β receptor (IFNAR) and the consequent activation of the downstream signalling [77]. Additionally, HPV16 E7 targets p48 (also known as IRF9) impairing the translocation to the nucleus of ISGF3, a heterodimer formed by STAT1-STAT2-p48, and consequential activation of antiviral genes expression [86,126,127].

Microarray analysis of HPV31 or HPV16 infected-keratinocytes showed a decrease in STAT1 transcription promoted by E6 and E7 [79,80,92]. Like STAT1, several other ISGs were shown to be downregulated upon HPV31, HPV18 and HPV16 infection [79,80]. PKR (an IFN-inducible protein that recognizes dsRNA, activating IFNs expression, and shuts-down host transcription) was shown to have its transcription impaired during HPV infection, and to be re-localized from the cytosol to P-bodies by E6 [78,128]. The same downregulation on transcription induced by E6 and E7 has been reported in different studies for several PRRs, such as TLR3, TLR9 and RIG-I [48,80,90,129,130]. The mechanism proposed for E7-mediated downregulation of TLR9 transcription was through histone modifications [90,91]. HPV16 and HPV18 E2 were even associated with the decrease in STING and IFN-κ transcription [68]. Moreover, this was also observed in clinical samples of low-grade CIN [68].

HPV E7 also impairs antiviral genes transcription through the induction of host DNA methylation by DNMT1, a transcription repressor [87]. Transcription of the chemokine CXCL14, essential for leukocyte attraction to the infection site, is affected by this process. HPV E6 was also associated to DNA methylation of the IFN-κ gene [112].

Curiously, IFNs have been used as therapy in clinical cases with HPV lesions, and non-responsiveness to IFNs was associated with higher levels of E7 protein [131].

## 5. Cellular Innate Immunity and Cancer Progression in HPV Infection

**Figure 4:**
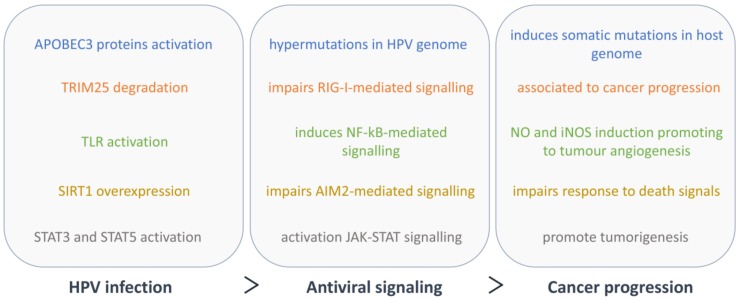
HPV evasion of the antiviral signalling and corresponding impact on carcinogenesis.

As previously mentioned, malignant transformation is an unwanted consequence for HPV, as it implies a lack of production of new virions. This event is rather a consequence of the unspecific targeting of HPV E6 and E7, whose sequences get integrated into the host genome while losing their viral transcription regulator. Besides modifying cell growth, differentiation and genome stability processes, these proteins alter specific cellular antiviral response mechanisms that have been associated with cancer progression and have a critical role in inflammatory processes and tumorigenesis (summarized in Figure 4).

APOBEC3 proteins exert their antiviral function through the induction of hypermutations in the HPV genome [60,61]. They may furthermore contribute to cancer mutagenesis by inducing somatic mutations in host genome [61]. Similarly, SIRT1 overexpression induced by HPV impairs AIM2-mediated immunity, and this inhibition allows HPV-infected cervical cancer cells to escape death and continue their growth. Moreover, SIRT1 expression in HPV-infected cervical cancers was associated with a poor clinical outcome [123].

Additionally, as TRIM25 has been associated to cancer–related pathways, its targeting by E6 may also regulate other functions of this protein that should be addressed in the context of HPV-associated cancer progression [132].

The influence of HPV on the inflammatory response is still controversial. While most studies using cell lines show an inhibition of the NF-κB pathway, in CIN and cervical cancer, HPV seems to induce the expression of inflammatory cytokines, which correlates with cancer progression [124,125]. A recent study has shown an increase of nitric oxide (NO) and inducible NO synthase (iNOS), which was suggested to be mediated by TLR-induced NF-κB signalling, in cervical samples from HR-HPV-infected patients [133]. Since NO is a critical component of the tumour microenvironment and promotes tumour angiogenesis, as well as tumour cell invasion and metastasis, it has been studied as a possible target for cancer therapy [134].

Contrary to the mentioned components of JAK/STAT, which are inhibited during viral infection, HPV also modulate STAT3 and STAT5, respectively, by increasing their activity. STAT3 stimulation leads to cell cycle progression and cell survival, suggesting its importance in the life cycle of HPV18 [135,136,137,138,139]. Likewise, STAT5 activation induces genome amplification in differentiating cells, through the exploitation of the DNA damage response [140]. Both STAT3 and STAT5 have been extensively studied in the context of tumorigenesis [141,142]. Moreover, it has been suggested that STAT3 expression correlates with increased severity of HPV lesions, being a possible target for therapy [139].

## 6. Conclusions

The antiviral defence mechanisms that recognize HPV early in infection are still poorly elucidated and most of what is known was inferred from HPV proteins’ overexpression studies. Nonetheless, several reports have demonstrated that different HPV efficiently evade the cellular antiviral signalling pathways using diverse strategies throughout their life cycle.

**Figure 5:**
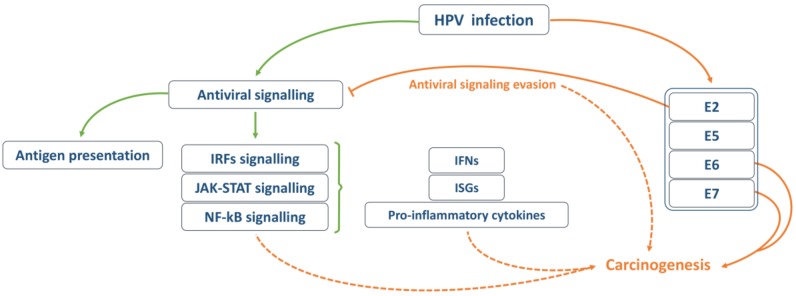
Overview of the interplay between HPV and the cellular antiviral signalling and its impact on carcinogenesis. Orange arrows represent HPV-mediated events, while green arrows represent host defence processes.

The oncogenes E6 and E7 are the viral proteins most involved in immune evasion, targeting almost all cellular innate immune pathways in a synergetic manner. However, most of the evasion mechanisms reported for HPV have been observed in vitro, and whether these results can be translated to the clinic remains unknown. The effectiveness of these studies is highly impaired by the fact that the interplay between HPV and their host cells changes during the different cell differentiation stages and disease progression. Nonetheless, it has been shown that E6 and E7 levels are inversely correlated to IFN treatment response and, more importantly, as discussed above, many of these evasion strategies directly correlate to the development of HPV-induced tumorigenesis (Figure 5).

Furthermore, although prophylactic vaccines are effective in averting infection of the most medically relevant HR-HPV, they do not exert any effect on existing infections [143]. Hence, it is essential to further analyse and understand the mechanisms behind HPV evasion of the cellular innate immunity and their correlation to HPV-induced persistence and tumorigenesis. These studies may reveal essential to the discovery of new cellular targets for the development of novel antiviral and anticancer therapies.

## Figures and Tables

**Table 1:** Human papillomavirus (HPV) types and associated lesions. Represented in bold are the most frequent high-risk HPV. HPV types that are not yet fully established as high-risk are represented between brackets.

Group	Type of Lesions	HPV Types
High-risk	Intraepithelial neoplasia and cervical cancer	**16, 18**, 31, 33, 35, 39, 45, 51, 52, 56, 68, 73, 82 (26, 53, 66)
Low-risk	Intraepithelial neoplasia or genital warts	6, 11, 40, 12, 43, 44, 53, 54, 61, 72, 73, 81

**Table 2:** HPV proteins’ functions on the evasion of the cellular antiviral response.

Viral Protein	HPV Type	Effects on Innate Immunity
E2	HPV16 HPV18	impairs transcription of the stimulator of IFN genes (STING) and IFN-κ, and their downstream antiviral genes [68]
E5	HPV16	inhibits the human leukocyte antigen class I (HLA-I) transport, decreasing its surface expression [69,70,71]
E6	HPV16	induces degradation of pro-IL-β through the ubiquitin ligase E6-AP [72]
	HPV16	induces the tripartite motif-containing protein 25 (TRIM25) degradation suppressing the retinoic acid-inducible gene-I (RIG-I)-mediated expression of IFN-β, chemokines, and ISGs [73]
	HPV16	binds to IRF3, inhibiting its transcription activities [74]
	HPV16	reduces transcription activity of CBP/p300, and consequentially NF-κB promoter activity [75,76]
	HPV18	binds to the tyrosine-protein kinase (TYK2), inhibiting the downstream signalling [77]
	HPV16 HPV31	re-localizes the protein kinase R (PKR) to P-bodies, impeding the downstream signalling [78]
	HPV31 HPV16 HPV18	inhibits STAT1 binding and ISGs transcription [79,80]
E7	HPV16	induces the overexpression of endoplasmic reticulum aminopeptidase 1 (ERAP1) decreases epitopes presentation [81]
	HPV16 HPV11 HPV18	binds to IRF1, inhibiting its promoter activity through histone deacetylases (HDACs) [82,83,84]
	HPV16 HPV18	binds to the NF-κB kinase (IKK) complex, impairing NF-κB signalling [75]
	HPV16	impairs DNA binding activity of NF-κB, through impairment of p65 subunit functions [82,85]
	HPV16	binds to p48, inhibiting the IFN-stimulated gene factor 3 (ISGF3)-mediated gene expression [86]
	HPV16	binds to the DNA methyltransferase (DNMT1), impairing antiviral gene transcription through epigenetic modification [87]
	HPV18	binds to STING, impairing IFNs and pro-inflammatory cytokines expression [88]
	HPV18	induces of the H3K9 methyltransferase (SUV39H1) transcription, which promotes epigenetic silencing of PRRs [89]
	HPV16 HPV38	inhibits of TLR9 promoter region through the formation of a inhibitory complex and induction of HDACs [90,91]
E6 E7	HPV16	competes with the transcription promoters of interleukin-18 (IL-18), impairing its expression [85]
E6/E7	HPV16	inhibit of STAT1 binding to DNA and antiviral genes transcription [92]
E6/E7	HPV16	induce the overexpression of p100 and p105 and their re-localization, which inhibit the transcriptional activity of NF-κB [93]
E6/E7	HPV38	decrease MHC-I (human HLA-I) expression through downregulation of STAT1 expression [94]
